# Discovery of Tricuspid Fibroelastoma on Echocardiography

**DOI:** 10.7759/cureus.17359

**Published:** 2021-08-22

**Authors:** Yesha Rana, Ramyashree Tummala, Krysthel Engstrom, Nina Kukar, Deepika Misra

**Affiliations:** 1 Department of Internal Medicine, Icahn School of Medicine at Mount Sinai, Mount Sinai Beth Israel Medical Center, New York City, USA; 2 Department of Cardiology, Icahn School of Medicine at Mount Sinai, Mount Sinai Beth Israel Medical Center, New York City, USA; 3 Department of Cardiology, Icahn School of Medicine at Mount Sinai, Mount Sinai West Hospital, New York City, USA

**Keywords:** cardiac tumor, papillary fibroelastoma, cardiac mri, echocardiography, benign cardiac tumor

## Abstract

Papillary fibroelastoma (PFE) is a benign cardiac tumor usually incidentally found on cardiac imaging. They are typically located on the left-sided heart valves and are concerning primarily due to their risk for embolization into the periphery. Right-sided PFE, however, is very rare and their management is not well known. We present a 66-year-old male with a past medical history of HIV on antiretroviral therapy presenting with new exertional dyspnea. Upon workup, he was found to have a mass on the tricuspid valve seen on echocardiography which was ultimately resected and found to be a tricuspid fibroelastoma. The clinical management of right-sided PFE is poorly documented. Treatment of PFE in an asymptomatic is dependent on characteristics such as location, mobility, and risk of embolization. Echocardiography has made the incidental diagnosis of PFE a common issue in asymptomatic patients such as ours.

## Introduction

Primary cardiac tumors are extremely rare, and over 75% of primary cardiac tumors are benign. Papillary fibroelastoma (PFE) is a rare, primary cardiac tumor that is more commonly associated with left heart cardiac valves and is most commonly incidentally found on echocardiography. These tumors typically have sizes that can range between 2 and 70mm, and over 80% of PFEs are typically found on left-sided heart valves [1,2]. Unlike left-sided cardiac tumors, management of right-sided cardiac tumors is unclear. We report a finding of a mass on echocardiography, later found to be tricuspid fibroelastoma after resection, in a patient presenting with dyspnea.

## Case presentation

A 66-year-old male with a past medical history significant for HIV diagnosed in 1995 managed on antiviral therapy, Kaposi’s Sarcoma of the right foot, diabetes mellitus, hypertension, hyperlipidemia, and hypothyroidism presented to the cardiology clinic for new-onset dyspnea over the last few months. He was primarily asymptomatic prior, but now he was only able to walk 40 blocks over 1 hour prior to getting fatigued. Otherwise, he denies having chest pain, palpitations, dizziness, orthopnea, or leg swelling. He also denied toxic habits and prior cardiovascular history. His vital signs and physical exam were unremarkable.

**Figure 1:**
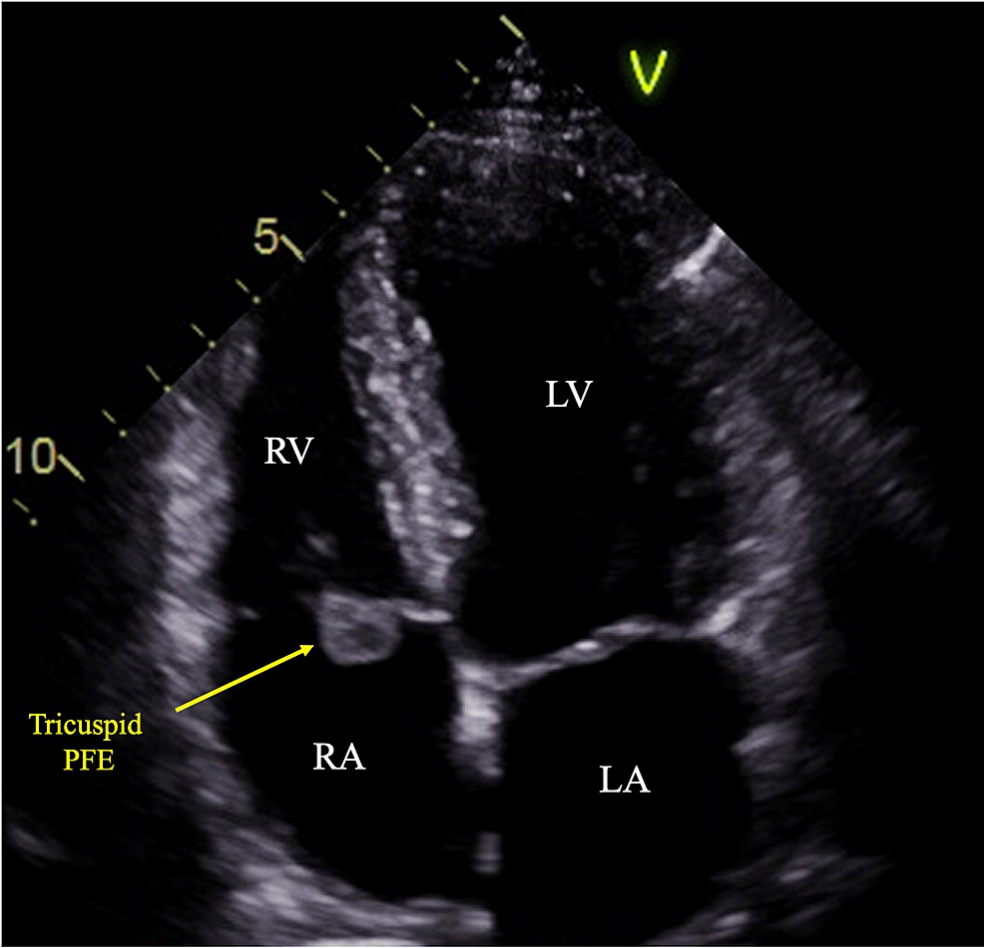
Apical four-chamber view on echocardiography visualizing a mass on the atrial side of the septal leaflet of the tricuspid valve. LA, left atrium; LV, left ventricle; RV, right ventricular; RA, right atrium; PPE, papillary fibroelastoma

Initial EKG showed normal sinus rhythm with no ST-T-wave abnormalities and Qtc 417 ms. An exercise transthoracic echocardiogram (TTE) was obtained due to exertional dyspnea and strong premature coronary artery disease history in his family. Resting imaging showed a left ventricular ejection fraction of 60%, normal left and right ventricular chamber size and function, and mildly dilated left atrium. The incidental finding of the stress TTE was the presence of a round, mobile echo-dense mass attached to the septal leaflet of the tricuspid valve (TV) on the atrial side measuring 1.7cm x 1.3cm with mild tricuspid regurgitation (TR) and a patent foramen ovale (PFO) concerning for vegetation or possible tumor (Figure 1).

**Figure 2:**
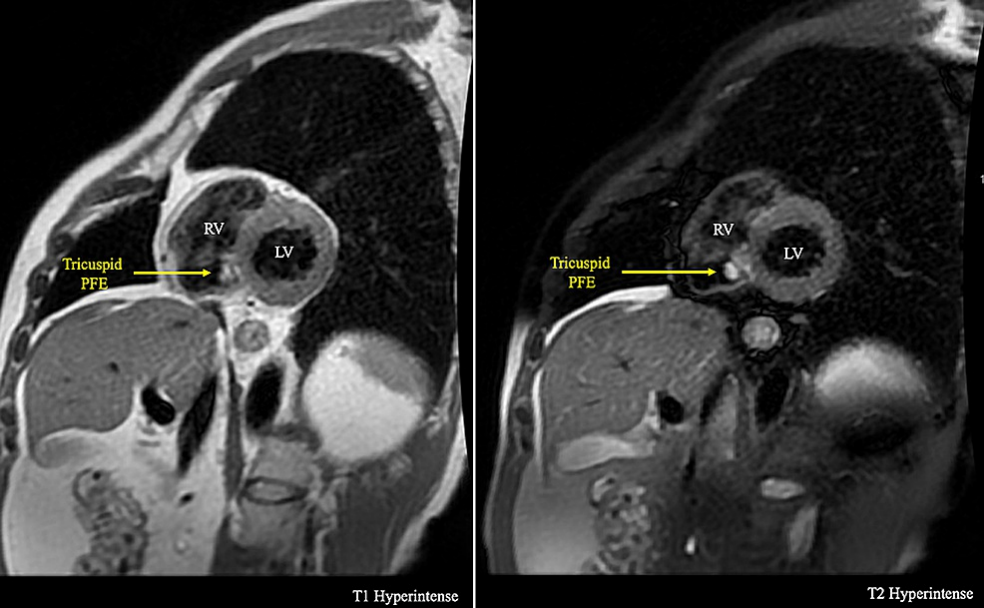
cMRI short-axis view showing T1 hyperintense (left) and T2 hyperintense (right) images depicting a cross-section showing a mass on the TV. cMRI, cardiac MRI; TV, tricuspid valve

**Figure 3:**
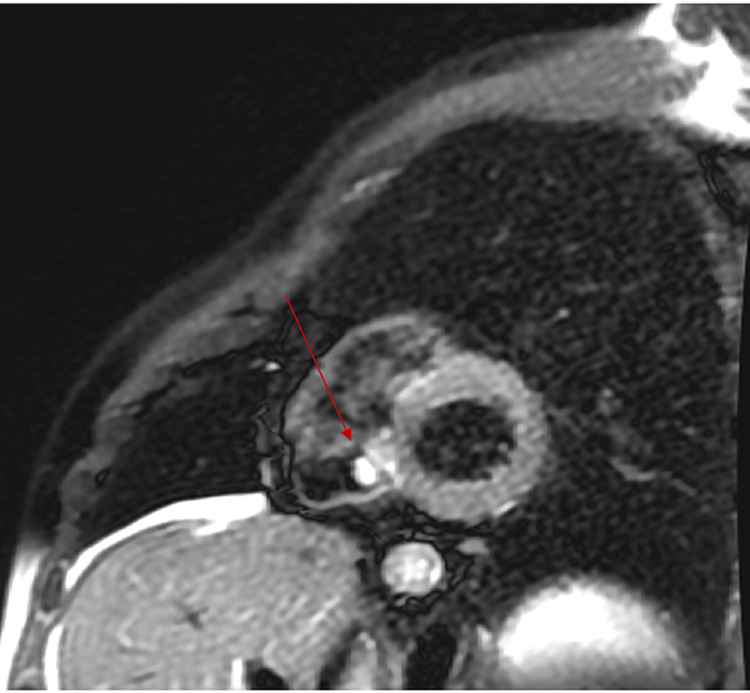
cMRI cine image depicting T2 hyperintensity showing a right-sided mass on the tricuspid valve.

Upon further workup and questioning, we found a lack of risk factors (i.e., IV drug use), normal white blood cell count, and no fever. Multiple sets of blood cultures were obtained at the same time from different locations were found to be all negative thus making infective endocarditis less likely. After negative infectious workup, a cardiac MRI (cMRI) to further characterize the right-sided mass showed a small mass on the atrial side of the septal leaflet of the TV measuring 0.9cm x 0.5cm in the short-axis orientation. The mass was found to be hyperintense on T2-weighted imaging, mildly hyperintense on T1-weighted imaging, and enhanced on post-contrast findings which are concerning for the presence of a tumor rather than vegetation or thrombus (Figures 2, 3).

The decision to resect the tumor was made after presurgical evaluation due to the high risk for embolization given the size of the mass and the presence of a PFO. Left heart catheterization was unremarkable and the carotid Doppler study was significant for mild to moderate bilateral carotid disease. He underwent successful complete excision of mass without causing damage to the TV as well as successful closure of PFO. The post-surgical course was uncomplicated, and he was discharged three days postoperatively. Finally, Surgical pathology confirmed the presence of a fibroelastoma. On follow-up, the patient reported continued improvement in symptoms and was feeling better overall.

## Discussion

PFE is a rare benign cardiac tumor that predominantly affects the cardiac valves particularly on the left side of the heart. Although its true prevalence is unknown due to its asymptomatic presentation and incidental discoveries, recent studies have reported a prevalence of about 10%, with tricuspid PFE being reported to be 6%-15% [1-3]. Mean age of detection is about 60 years because of the usefulness of echocardiography in the older population. The etiology of PFEs remains largely unknown due to their rare occurrence. They have been theorized to mainly originate from coalescing microthrombi in areas of minor endothelial damage on the valvular surface [4]. The PFEs can continue to grow and have the potential of embolizing causing a multitude of complications ranging from stroke, myocardial infarction, pulmonary embolism, or even sudden cardiac death [4]. They are typically found incidentally on imaging. In our case, it is not quite clear if the tricuspid PFE was the only cause of our patient’s dyspnea; however, it could be stated that the PFE likely contributed to the manifestation of his symptoms as they improved after resection. 

Cardiac masses could have a number of different etiologies ranging from infectious processes, thrombi, and primary or secondary malignancies. Therefore, it is important to have multiple imaging modalities that can help supplement clinical data in determining the possible causes of the mass. The portability, wide availability, lack of radiation, and relatively low cost make echocardiography easily accessible. Echocardiography of our patient was able to show a round, mobile, echo-dense mass leading to further investigation in our patient. PFEs generally range from 2mm to 40mm and are usually found to be pedunculated [5]. They can also originate on the atrial or ventricular side of the valve. Echocardiography, unfortunately, only provides a little information warranting further evaluation and imaging.

CMRI can supplement TTE findings to further differentiate the makeup of the mass. Neoplastic cells have been found to be larger than normal cells thus containing more intracellular water, which leads to prolonged T1/T2 relaxation times thus allowing cMRI to pick up the inherent differences between tumors and normal tissue [6]. The gold standard for diagnosis of the tumor requires cMRI to differentiate it from other echogenic masses by way of the larger field of view, better special resolution and tissue characterization, lack of attenuation, and ability to image at any prescribed plane, allowing us to differentiate between tumor and thrombus [4,7].

Ultimately, a definitive diagnosis is made on histopathology after surgical resection or biopsy. Histopathology of PFEs typically shows a central core of fibrin, mucopolysaccharide-rich connective tissue surrounded by a layer of the endothelium [5,8]. Although this is the case, decisions concerning surgical resection of a PFE in an asymptomatic patient depend primarily on the size, location, mobility of the tumor, and risk of complications such as embolization. Furthermore, excision of solitary right-sided PFE in asymptomatic patients remains controversial primarily because of how rare the right-sided tumors are. Regardless, surgery is indicated for large, mobile tumors. Also, the presence of a PFO, such as in our patient, may play an important role in a decision favoring surgery.

The prognosis of PFE is good following the excision of the tumor especially because of its benign nature and rare recurrence after resection [2]. Removal of the tumor also prevents major complications particularly those due to embolization. There is still limited knowledge about the management of right-sided PFE. Potentially, minimally invasive surgery may be an option in the future for uncomplicated cases thus minimizing the need for a sternotomy and scarring for patients. More research is needed to manage right-sided PFE.

## Conclusions

Right-sided PFEs are rare and often found incidentally on cardiac imaging, especially echocardiography. Although surgical resection has been clearly shown to improve morbidity and mortality in left-sided tumors, management of right-sided tumors continues to be unclear. Further research is necessary to create better management guidelines for the management of right-sided tumors. This case presents an interesting report of right-sided PFE with surgical resection and pathological confirmation.
